# Lyme disease bacterium does not affect attraction to rodent odour in the tick vector

**DOI:** 10.1186/s13071-015-0856-8

**Published:** 2015-04-28

**Authors:** Jérémy Berret, Maarten Jeroen Voordouw

**Affiliations:** Laboratory of Ecology and Evolution of Parasites, Institute of Biology, University of Neuchâtel, Rue Emile-Argand 11, 2000 Neuchâtel, Switzerland

**Keywords:** *Borrelia burgdorferi*, *Borrelia afzelii*, *Borrelia garinii*, Host choice behaviour, Host manipulation, *Ixodes ricinus*, Lyme borreliosis, Tick questing behaviour, Tick-borne disease, Vector-borne pathogen

## Abstract

**Background:**

Vector-borne pathogens experience a conflict of interest when the arthropod vector chooses a vertebrate host that is incompetent for pathogen transmission. The qualitative manipulation hypothesis suggests that vector-borne pathogens can resolve this conflict in their favour by manipulating the host choice behaviour of the arthropod vector.

**Methods:**

European Lyme disease is a model system for studying this conflict because *Ixodes ricinus* is a generalist tick species that vectors *Borrelia* pathogens that are specialized on different classes of vertebrate hosts. Avian specialists like *B. garinii* cannot survive in rodent reservoir hosts and vice versa for rodent specialists like *B. afzelii*. The present study tested whether *Borrelia* genospecies influenced the attraction of field-collected *I. ricinus* nymphs to rodent odours.

**Results:**

Nymphs were significantly attracted to questing perches that had been scented with mouse odours. However, there was no difference in questing behaviour between nymphs infected with rodent- versus bird-specialized *Borrelia* genospecies.

**Conclusion:**

Our study suggests that the tick, and not the pathogen, controls the early stages of host choice behaviour.

**Electronic supplementary material:**

The online version of this article (doi:10.1186/s13071-015-0856-8) contains supplementary material, which is available to authorized users.

## Background

Many tick species appear to be generalists that feed on a wide range of vertebrate hosts [1-3]. The broad host range of generalist tick species has important consequences for the ecology of tick-borne pathogens and the human risk of contracting tick-borne infections [4]. In Europe, for example, *Ixodes ricinus* is a generalist tick that exposes many vertebrate species (including humans) to a wide variety of tick-borne diseases including Lyme borreliosis and tick-borne encephalitis. From the perspective of the tick-borne pathogen, not all hosts are created equal because vertebrate species can differ substantially in their transmission competence [5,6]. When the tick vector preferentially feeds on pathogen-incompetent hosts, host choice can be a source of conflict between the tick and the pathogen. This conflict is illustrated by the western blacklegged tick, *Ixodes pacificus*, and the tick-borne bacterium, *Borrelia burgdorferi*. The tick prefers lizards to rodents to obtain a blood meal [7-9]. In contrast, the pathogen is killed by lizard blood [10] and prefers the highly competent rodent reservoir host. Thus the conflict over host choice can be a question of life and death for the tick-borne pathogen.

The qualitative manipulation hypothesis suggests that vector-borne parasites can resolve this conflict in their favour by manipulating the host choice behaviour of the arthropod vector [11,12]. Vector-borne pathogens can manipulate the biting behaviour of their arthropod vectors to increase pathogen transmission [13-20]. Similarly, vector-borne pathogens can manipulate the odour profile of the vertebrate host to make them more attractive to passing vectors [13,21-24]. Thus vector-borne pathogens are manipulative but to date there is not much evidence that vector-borne pathogens can manipulate the vector’s selection of the vertebrate host. There are some recent reports that tick-borne pathogens can influence host choice behaviour in *I. ricinus* ticks [25,26]. This preliminary work motivated us to investigate whether tick-borne *Borrelia* pathogens can manipulate host choice behaviour in *I. ricinus* ticks to maximize their transmission success.

The European system of Lyme borreliosis is a model system for testing whether vector-borne pathogens can manipulate host choice behaviour in the arthropod vector. The vector, *I. ricinus*, is a generalist tick that feeds on mammals, birds, and lizards [2]. This tick is responsible for transmitting a diversity of spirochete bacteria belonging to the *B. burgdorferi* sensu lato (s. l.) genospecies complex [27]. Members of this genospecies complex have specialized on different classes of vertebrate hosts. *Borrelia afzelii*, *B. burgdorferi* sensu stricto (s. s.), and *B. bavariensis* are specialized on rodent reservoir hosts [28-34] whereas *B. garinii* and *B. valaisiana* are specialized on avian reservoir hosts [29-31,35-42]. The mechanism of this host specialization appears to be mediated by the complement system of the vertebrate host [30,31]. *Borrelia afzelii* is killed by the complement system of birds and conversely, *B. garinii* is killed by the complement system of rodents [30,31]. Thus the complement system of the wrong vertebrate host is the type of existential threat that should exert strong selection on *Borrelia* pathogens to evolve manipulation of host choice behaviour in *I. ricinus* ticks.

The purpose of this study was to test whether infection with *B. burgdorferi* s. l. pathogens influenced the host searching behaviour (or questing behaviour) of *I. ricinus* nymphs. We focussed on the nymphal ticks because this stage is responsible for infecting the rodent and avian reservoir hosts with the corresponding *Borrelia* pathogens [27]. In contrast, adult *I. ricinus* ticks mostly feed on large vertebrates like deer that are incompetent hosts for *Borrelia* pathogens. We predicted that ticks infected with rodent-specialized genospecies would be attracted to rodent odours whereas ticks infected with bird-specialized genospecies would avoid such odours. To our knowledge, this is the first test of the qualitative manipulation hypothesis in a Lyme disease system. Manipulation of host choice behaviour in *I. ricinus* by *Borrelia* pathogens will have important implications for our understanding of the epidemiology of Lyme disease [4].

## Methods

### Sampling wild *Ixodes ricinus* ticks

Wild *I. ricinus* ticks were sampled between March and May 2014. The sampling sites were located above Neuchâtel, Switzerland (47°00 N, 6°56 E, ~725 m above sea level) and consisted of mixed forest dominated by deciduous trees. We captured ticks by dragging a white cotton flag over the vegetation. Nymphal ticks were kept in groups of 20 in glass tubes that were stored in plastic boxes containing a layer of water to ensure high relative humidity (~98%). The boxes were kept in the laboratory under ambient conditions.

### Description of the tick questing behaviour apparatus

The tick questing behaviour apparatus gave the *I. ricinus* nymphs a choice of selecting one of eight questing perches. The questing perches consisted of glass rods (diameter = 0.2 cm, length = 20 cm) that were oriented in the vertical plane by sinking the bottom 2 cm of each rod in a block of floral foam. The eight glass rods were arranged in a circle (diameter = 7 cm) with a distance of ~2.5 cm between adjacent rods. A cone made of Whatman filter paper (diameter of filter paper = 9 cm, cone circumference = 7 cm, cone height = 3 cm) was placed in the middle of the circle of glass rods with the pointy side (apex) down. The apex of the cone was in contact with the floral foam whereas the base of the cone was in contact with each of the eight glass rods. During a trial, nymphs were placed in the apex from where they ascended the walls of the cone to select one of the eight questing perches. The distance from the apex to each of the eight glass rods was 4.5 cm. A layer of Vaseline was placed around the floral foam to trap any nymphs that climbed out of the filter cone.

### Description of the tick questing behaviour trials

To capture the odours of the rodent reservoir host, a piece of medical gauze was left overnight in a cage containing a single BALB/c mouse [43]. This scented piece of medical gauze was attached to one of the eight questing perches. Similar-sized pieces of medical gauze without odours were attached to the seven other questing perches. Each questing behaviour trial consisted of emptying a tube of 20 wild *I. ricinus* nymphs in the apex of the cone. Some trials had fewer than 20 nymphs because some nymphs had died inside the tube. Nymphs were given 90 minutes to choose one of the eight questing perches. After 90 minutes, each nymph was recorded as being in one of three different states: (1) missing nymphs that had climbed out of the cone and left the system, (2) inactive nymphs that had not left the filter paper cone, and (3) active nymphs (or questing nymphs) that had ascended one of the eight questing perches. The nymphs were put in individual Eppendorf tubes and frozen at -80°C for retrospective analysis of their *B. burgdorferi* s. l. infection status.

Four types of trials, hereafter referred to as A, B, C, and D, were conducted that differed with respect to the collection dates of the wild *I. ricinus* nymphs and the source of the rodent odour (Table 1). In trial type A, nymphs were collected in March 2014 and the focal piece of medical gauze was scented with odours from uninfected BALB/c mice (10 trials). In trial types B, C, and D, the nymphs were collected in late April and early May 2014, and the focal piece of medical gauze was scented with (B) odours from uninfected BALB/c mice (10 trials), (C) odours from BALB/c mice that had been experimentally infected with *B. afzelii* (10 trials), and (D) no mouse odour (10 trials). Thus a total of 40 trials were conducted with 20 ticks per trial (total = 800 ticks). To incorporate variation in odour profile between BALB/c mice, a different mouse was used for each trial (20 uninfected female mice and 10 *B. afzelii*-infected female mice). To avoid position effects on tick host choice, the position of the scented questing perch was changed at random between trials. The trials took place in a darkened room between the hours of 10:00 and 16:00 over a period of ten weeks (April 4 to June 10, 2014).

**Table 1:** **The four different types of tick questing behaviour trials**

**Type**	**Date**	**Source of odour**	**Mice**	**Trials**	**Ticks**
A	18/03, 28/03	Uninfected mice	10	10	20
B	24/04,06/05	Uninfected mice	10	10	20
C	24/04, 06/05	*B. afzelii*-infected mice	10	10	20
D	24/04, 06/05	None	0	10	20

The four different trial types were labelled A, B, C, and D. The tick collection date, source of odour, number of mice, number of trials, and the number of ticks per trial are shown.

### Ethical approval

All experiments involving mice respected the Swiss legislation on animal experimentation and were authorized by the Veterinary Service of the Canton of Neuchâtel (Authorization number NE01/13). The mice that had been experimentally infected with *B. afzelii* were from another experiment (Authorization number NE2/2012).

### *Borrelia burgdorferi* s. l. infection status of wild *I. ricinus* ticks

Quantitative PCR (qPCR) was used to determine the *B. burgdorferi* s. l. infection status of the wild *I. ricinus* nymphs. A reverse line blot (RLB) assay was used to determine the identity of the *Borrelia* genospecies. The RLB assay allowed us to identify the six most common *B. burgdorferi* s. l. genospecies in Switzerland: *B. afzelii*, *B. bavariensis*, *B. burgdorferi* s. s., *B. garinii*, *B. lusitaniae*, and *B. valaisiana*. Total DNA was extracted from the nymphs using a TissueLyser II and DNeasy 96 Blood & Tissue kit well plates following the manufacturer’s instructions [44].

A quantitative PCR amplifying a 132 base pair fragment of the *flagellin* gene [45] was used to detect and quantify *Borrelia* DNA. The 20 μl qPCR mixture consisted of 10 μl of 2x Master Mix (FastStart Essential DNA Probes Master, Roche Applied Science), 3 μl of water, 0.4 μl of 20 μM primer FlaF1A, 0.4 μl of 20 μM primer FlaR1, 0.2 μl of 10 μM Flaprobe1, and 5 μl of DNA template. The thermocycling conditions included a denaturation step at 95°C for 10 min followed by 55 cycles of 60°C for 30 sec and 95°C for 10 sec using a LightCycler® 96 (Roche Applied Science, Switzerland).

Of the 800 nymphs, 12 had died before the trials and the remaining 788 tick DNA extractions were processed in 31 different 96-well qPCR plates. Each qPCR plate contained 28 tick DNA extractions, 3 standards, and 1 negative control (distilled water), all run in triplicate, for a total of 96 qPCR reactions. The standards consisted of the pB31/41-9 plasmid containing a single copy of the *flagellin* gene that had been transformed into competent *E. coli* cells [46]. A mini-prep of this plasmid was diluted so that the three standards contained 14,000, 1,400 and 140 copies of the *flagellin* gene, respectively. The LightCycler® 96 software (Roche Applied Science, Switzerland) calculated the standard curves and the absolute number of spirochetes present in each positive sample.

The RLB assay amplified the variable spacer region between two repeated copies of the 23S and 5S ribosomal genes [47]. The protocol for this RLB assay has been described elsewhere [48]. In cases where the RLB failed, Sanger sequencing of the *RecA* gene was used to identify the *Borrelia* genospecies. A 156 base pair fragment of the *RecA* gene was amplified as described elsewhere [49]. The amplicons were purified using the MSB® SPIN PCRAPACE kit from STRATEC Biomedical AG (Birkenfeld, Germany) and sequenced by Microsynth AG (Balgach, Switzerland). The *RecA* gene sequences were blasted on NCBI [50] to determine the identity of the *Borrelia* genospecies.

### Statistical analysis

All the statistical analyses were performed in RStudio© [51]. The 95% confidence limits of all the proportions in the text and figures were calculated using the binom.test function in R.

### Tick questing activity and tick attraction to rodent odour

Ticks that had left the system by climbing out of the filter paper cone were classified as missing. The ticks that remained in the system at the end of the trial were classified as inactive or active. Inactive ticks had not left the filter paper cone whereas active ticks (or questing ticks) had selected one of the eight questing perches. For simplicity and in all that follows, the missing ticks were not included in the statistical analyses. For the ticks that remained in the system (missing ticks were excluded), tick questing activity was calculated as the proportion of ticks that were active. For the ticks that were active (missing ticks and inactive ticks were excluded), attraction to rodent odour was calculated as the proportion of active ticks that had selected the focal scented questing perch. Tick questing activity and tick attraction to rodent odour are both binomial response variables.

### Tick preference for the scented questing perch

To test whether questing nymphs preferred the questing perch scented with mouse odours, an exact binomial test was used for each of the 30 trials to calculate the probability that random chance could have produced the observed distribution of questing nymphs. For this exact binomial test, the null hypothesis of no preference was that each of the eight questing perches had a probability of 1/8 = 0.125 of being selected by the questing nymphs.

### Effect of mouse odour on tick questing activity and tick attraction to rodent odour

Generalized linear mixed effects models (GLMMs) with binomial errors were used to analyse the two binomial response variables: tick questing activity and tick attraction to rodent odour. The GLMMs were run in R using the ‘glmer’ function of the R package ‘lme4’. To test whether our method of capturing mouse odours was effective, tick activity was modelled as a function of the fixed factor mouse odour with three levels: no mouse odour, odour from uninfected control mice, and odour from *B. afzelii*-infected mice. Trial identity was used as a random factor.

### Effect of *Borrelia* infection in the tick on tick questing activity and tick attraction to rodent odour

To test whether infection with *B. burgdorferi* s. l. in the tick influenced tick questing activity and tick attraction to rodent odour, the ten trials with no mouse odour (trial type D) were excluded from the statistical analysis. This analysis also combined the trials for trial types A, B and C because the previous analysis had found no effect of mouse infection status on tick questing activity. The two binomial response variables were modelled as a function of one of three fixed factors: *Borrelia* genospecies, *Borrelia* ecotype, and *Borrelia* infection. The levels of the *Borrelia* genospecies factor were combined to create the *Borrelia* ecotype and *Borrelia* infection factors. The *Borrelia* genospecies had five levels: uninfected, *B. afzelii*, *B. burgdorferi* s. s., *B. garinii*, and *B. valaisiana*. The *Borrelia* ecotype factor had three levels: uninfected, rodent specialists (*B. afzelii*, *B. burgdorferi* s. s.), and bird specialists (*B. garinii*, *B. valaisiana*). The *Borrelia* infection had two levels: uninfected or infected with *B. burgdorferi* s. l. pathogens. Trial identity was used as a random factor.

## Results

### Missing ticks and active ticks

Of the 788 nymphs, there were 243 missing nymphs that left the system and 545 nymphs that remained in the system. Of the 545 nymphs in the system, 222 nymphs were inactive and 323 nymphs were active. A chi-square test of independence was used to test whether a tick’s decision to leave the system was influenced by its infection status and/or the identity of the *Borrelia* genospecies. This test confirmed that infection status and *Borrelia* genospecies did not influence the probability of whether the tick left the system (χ^2^ = 4.043, df = 5, p = 0.543). The 243 missing ticks were excluded from all subsequent statistical analyses.

### Effect of mouse odour on tick questing activity

Tick questing activity was higher in the trials with mouse odour than in trials without mouse odour. The mean tick questing activity was 1.7 times higher in the trials with mouse odour (trial types A, B, and C; n = 30 trials; 278 active ticks/428 total ticks; mean = 64.95%; 95% confidence limits (CL) = 60.22–69.47%) than in the trials without mouse odour (trial type D; n = 10 trials; 45 active ticks/117 total ticks; mean = 38.46%; 95% CL = 29.62–47.91%). The effect of mouse odour on tick questing activity was statistically significant (Δ df = 1, Δ dev = 11.496, p = 0.001).

For the 30 trials that used mouse odours, the mean tick questing activity was 73.58% (n = 10 trials; 117 active ticks/159 total ticks; 95% CL = 66.02–80.25%) for trial type A with March nymphs and odours from uninfected mice, 58.16% (n = 10 trials; 82 active ticks/141 total ticks; 95% CL = 49.56–66.40%) for trial type B with April nymphs and odours from uninfected mice, and 61.72% (n = 10 trials; 79 active ticks/128 total ticks; 95% CL = 52.72–70.17%) for trial type C with April nymphs and odours from *B. afzelii*-infected mice. There was no effect of *B. afzelii* infection in the mice on tick questing activity (Δ df = 2, Δ dev = 4.128, p = 0.127).

### Tick preference for the scented questing perch

**Figure 1:**
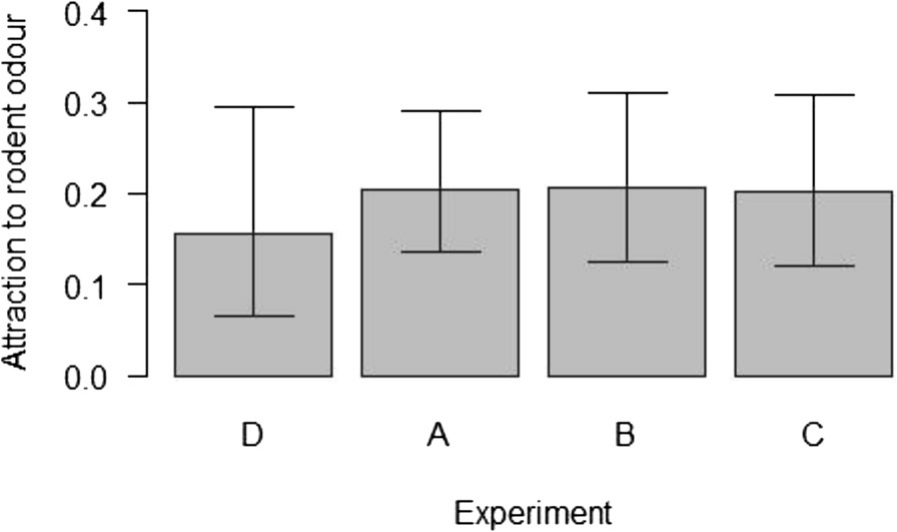
Proportion of active nymphs that chose the scented focal stick for the four trial types. The proportion of active *I. ricinus* nymphs that chose the focal stick scented with mouse odours is shown for each of the four trial types. The four trial types were: **(A)** March nymphs and odour from uninfected control mice (n = 10 trials), **(B)** April nymphs and odour from uninfected control mice (n = 10 trials), **(C)** April nymphs and odour from *B. afzelii*-infected mice (n = 10 trials), and **(D)** April nymphs and no mouse odour (n = 10 trials). Shown are the means and the 95% confidence limits.

The active nymphs were more likely to select the focal perch when it was scented with mouse odours (trial types A, B, C) than when it was unscented (trial type D; Figure 1). Across the 30 trials that used mouse odours, there were 278 active ticks of which 57 selected the scented questing perch. The percentage of active ticks that selected the scented questing perch (20.50% = 57/278; 95% CL = 15.91–25.73%) was 1.64 times greater than the null expectation (12.5% = 1/8) and this difference was statistically significant (two-sided binomial test; p < 0.001).

Of the 30 trials that used mouse odours, there were 11 trials where a significantly greater proportion of active nymphs selected the scented questing perch than expected from random chance alone (see Additional file 1). When the type I error rate is set at 5%, the probability of obtaining 11 type I errors in 30 trials is very low (p <0.000001). The active nymphs therefore had a strong preference for the scented questing perch.

### Correspondence between the qPCR and the RLB assay

The qPCR worked well as all the positive and negative controls tested positive and negative, respectively. The qPCR detected 223 infections with *B. burgdorferi* s. l. and was more sensitive than the RLB assay, which detected 206 infections. The correspondence between the two detection methods was high. The RLB detected 88.34% (197/223) of the infections detected by the qPCR and conversely, the qPCR detected 95.63% (197/206) of the infections detected by the RLB. The Pearson’s correlation between the two detection methods was positive and highly statistically significant (r = 0.883, t = 54.51, df = 786, p < 0.001). There were 26 ticks that were infected according to the qPCR but for which the RLB and Sanger sequencing of the *RecA* gene were unable to determine the *Borrelia* genospecies. These ticks were excluded from the statistical analysis.

### Identification of *Borrelia burgdorferi* s. l. genospecies in wild *I. ricinus* nymphs

Of the 788 *I. ricinus* nymphs, the RLB assay detected 197 single and 9 double infections with *B. burgdorferi* s. l. pathogens. The 197 single infections contained the following five *Borrelia* genospecies: *B. afzelii* (n = 22), *B. burgdorferi* s. s. (n = 4), *B. garinii* (n = 127), and *B. valaisiana* (n = 40), and unidentified *B. burgdorferi* s. l. (n = 4). No single infections with *B. lusitaniae* and *B. bavariensis* were detected in this study. The 9 double infections included: *B. afzelii* and *B. bavariensis* (n = 2), *B. afzelli* and *B. burgdorferi* s. s. (n = 1), *B. garinii* and *B. lusitaniae* (n = 5), and *B. garinii* and *B. valaisiana* (n = 1). For the analysis, these doubly infected ticks were treated as being singly infected with either *B. afzelii* (n = 3) or *B. garinii* (n = 6).

### Effect of *Borrelia* ecotype on tick questing activity and tick attraction to rodent odour

To test the effect of *Borrelia* ecotype on tick questing activity or tick attraction to rodent odour, the ten unscented trials (trial type D) were excluded from the statistical analysis. There was no significant difference in the explanatory power between the *Borrelia* genospecies factor and the *Borrelia* ecotype factor on tick questing activity (Δ df = 2, Δ dev = 3.291, p = 0.193) or on tick attraction to rodent odour (Δ df = 2, Δ dev = 0.538, p = 0.764). Thus the decision to combine *B. burgdorferi* s. s. and *B. afzelii* into a single ‘rodent specialist’ group and *B. garinii* and *B. valaisiana* into a single ‘bird specialist’ group was justified.

The mean tick questing activity was highest for the nymphs infected with the bird-specialized *Borrelia* ecotype (n = 76 active ticks/105 total ticks; mean = 72.38%; 95% CL = 62.80–80.66%; Table 2), intermediate for the uninfected nymphs (n = 179 active ticks/288 total ticks; mean = 62.15%; 95% CL = 56.28–67.78%; Table 2), and lowest for the nymphs infected with the rodent-specialized *Borrelia* ecotype (n = 13 active ticks/22 total ticks; mean = 59.09%; 95% CL = 36.35–79.29%; Table 2). However, there was no significant effect of *Borrelia* ecotype on tick questing activity (Δ df = 2, Δ dev = 2.919, p = 0.232).

**Table 2:** **Classification of nymphs according to** ***Borrelia*** **ecotype and tick questing activity state**

**(I) All trials**	**Missing**	**Inactive**	**Unscented**	**Scented**	**Total**
Uninfected	175	165	180	36	556
Rodent-specialist	5	10	11	3	29
Bird-specialist	53	39	65	16	173
Unidentified	10	8	10	2	30
Total	243	222	266	57	788
**(II) Trials A, B, C**	**Missing**	**Inactive**	**Unscented**	**Scented**	**Total**
Uninfected	116	109	150	29	404
Rodent-specialist	5	9	10	3	27
Bird-specialist	44	29	60	16	149
Unidentified	6	4	8	2	20
Total	171	151	228	50	600
**(III) Trials D**	**Missing**	**Inactive**	**Unscented**	**Scented**	**Total**
Uninfected	59	56	30	7	152
Rodent-specialist	0	1	1	0	2
Bird-specialist	9	10	5	0	24
Unidentified	4	4	2	0	10
Total	72	71	38	7	188

Nymphs were classified according to their *Borrelia* ecotype infection status and their state at the end of the tick questing behaviour trial. *Borrelia* ecotype infection status had four levels: uninfected, rodent-specialist (*B. afzelii*, *B. burgdorferi* s. s.), bird-specialist (*B. garinii*, *B. valaisiana*), and unidentified *B. burgdorferi* s. l. infection. The trial questing activity state had four levels: nymphs that had left the system (missing), nymphs that had not left the filter paper cone (inactive), nymphs that had selected an unscented questing perch (unscented), nymphs that had selected the focal scented questing perch (scented). (I) Nymphs are from all 40 trials (A, B, C, D). (II) Nymphs are from the 30 trials with mouse odour (trial types A, B and C). (III) Nymphs are from the 10 trials without mouse odour (trial type D).

**Figure 2:**
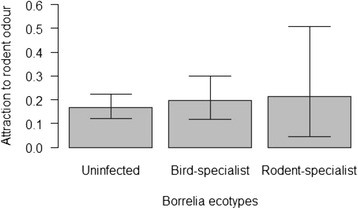
Proportion of active nymphs that chose the scented focal stick for each *Borrelia* ecotype. The proportion of active *I. ricinus* nymphs that chose the focal stick scented with mouse odours is shown for each of the three groups of nymphs. The three groups were: uninfected nymphs (16.20% = 29/179), nymphs infected with the bird-specialist ecotype (21.05% = 16/76), and nymphs infected with the rodent-specialist ecotype (23.08% = 3/13). The differences in attraction to rodent odour between the three groups of nymphs were not statistically significant. Shown are the means and the 95% confidence limits.

The preference for the focal perch scented with mouse odour was highest for the nymphs infected with the rodent-specialist ecotype (3 focal ticks/13 active ticks; mean = 23.08%; 95% CL = 5.04–53.81%), intermediate for the nymphs infected with the bird-specialist ecotype (16 focal ticks/76 active ticks; mean = 21.05%; 95% CL = 12.54–31.92%), and lowest for the uninfected nymphs (29 focal ticks/179 active ticks; mean = 16.20%; 95% CL = 11.12–22.43%; Figure 2). However, there was no significant effect of *Borrelia* ecotype on tick attraction to rodent odour (Δ df = 2, Δ dev = 0.983, p = 0.611).

### Effect of *Borrelia burgdorferi* s. l. infection on tick questing activity and tick attraction to rodent odour

There was no significant difference between the *Borrelia* ecotype and *Borrelia* infection status on tick questing activity (Δ df = 1, Δ dev = 0.932, p = 0.334) or on tick attraction to rodent odour (Δ df = 1, Δ dev = 0, p = 1). Thus the decision to combine all the *Borrelia* genospecies into a single infected group was justified.

The mean tick questing activity was 12.75% higher for the infected nymphs (89 active ticks/127 total ticks; mean = 70.08%; 95% CL = 61.32–77.88%) compared to the uninfected nymphs (179 active ticks/288 total ticks; mean = 62.15%; 95% CL = 56.28–67.78%). However, there was no effect of *Borrelia* infection on tick questing activity (Δ df = 1, Δ dev = 1.987, p = 0.158).

The mean preference for the scented perch was 31.77% higher for the infected nymphs (19 focal ticks/89 active ticks; mean = 21.35%, 95% CL = 13.37–31.31%) compared to the uninfected nymphs (29 focal ticks/179 active ticks; mean = 16.20%, 95% CL = 11.13–22.43%). However, there was no effect of *Borrelia* infection on tick attraction to rodent odour (Δ df = 1, Δ dev = 1.422, p = 0.233).

## Discussion

The present study found no evidence for the qualitative manipulation hypothesis in the European Lyme disease system [11,12]. Nymphs infected with rodent-specialized and bird-specialized *Borrelia* genospecies were equally attracted to the questing perches scented with rodent odours. Previous studies have shown that bird-specialized *Borrelia* genospecies are killed by the rodent complement system and are unable to establish systemic infections inside rodent reservoir hosts [30,31]. From the perspective of a bird-specialized *Borrelia* genospecies in a nymphal tick, biting a rodent reservoir host results in certain death and zero transmission success. We therefore expected *B. garinii* to be under strong selection to prevent nymphs from selecting rodent-scented questing perches but this was not the case. In our local Lyme disease system, immature *I. ricinus* ticks feed on rodents, birds, artiodactyls, and carnivores in the following frequencies: 28.0%, 16.6%, 40.0%, and 15.5% [52]. Random host choice will therefore kill 72.0% and 83.4% of the rodent-specialized and bird-specialized *Borrelia* infections, respectively. This analysis demonstrates that the generalist host choice of *I. ricinus* nymphs imposes a high source of mortality on the more specialized *Borrelia* pathogen. Despite this undesirable state of affairs, there was no evidence that *Borrelia* pathogens can manipulate attraction to rodent odour in *I. ricinus* nymphs.

A number of recent studies found suggestive evidence that tick-borne pathogens can influence host-seeking behaviour in *I. ricinus* ticks [25,26]. *Borrelia afzelii*-infected ticks did not respond to odours from accidental hosts (dogs and humans) whereas uninfected ticks responded to all odours [25]. Similarly, *I. ricinus* ticks infected with TBEV were attracted to the odours of competent rodent hosts but not to accidental hosts (dogs) [26]. More generally, infection with *Borrelia* pathogens is associated with a number of tick phenotypes that can affect the encounter rate between questing ticks and vertebrate hosts [48,53-58]. A major limitation of these correlative studies (including the present one) is the inability to establish a causal relationship between *Borrelia* infection and the observed phenotype. Future studies should use experimental infections to establish the pattern of causation between *Borrelia* infection and tick phenotype.

*Ixodes* ticks are ambush predators that position themselves on the vegetation and wait to encounter a vertebrate host [59]. The ability of *Ixodes* ticks to select ambush sites by using chemical cues left by passing hosts on the vegetation would have considerable adaptive value [60]. Previous experimental work has shown that the glandular secretions of deer contain kairomones that are attractive to adult *I. scapularis* ticks [60,61]. Other studies on *I. scapularis* found that deer urine was attractive to adult ticks whereas mouse urine was not attractive to immature ticks [62,63]. Our experimental approach stimulated nymph questing activity and allowed nymphs to identify and select the scented questing perch. One advantage of this method is that it is less invasive than using live hosts, which may not support the stress of participating in a host choice experiment [8].

Lees [64] divided the host selection by ambush-type ticks into three stages: (1) the tick selects an ambush site where it is likely to encounter a host, (2) the tick encounters and climbs on the host, and (3) the tick either rejects the host or inserts its feeding apparatus. In the present study, we only investigated the first stage and so it is possible that *Borrelia* pathogens manipulate the later stages of host selection. Future studies should test whether *B. garinii* can avoid death by preventing *I. ricinus* nymphs from attaching to rodent hosts and conversely, whether *B. afzelii* can block nymphs from attaching to avian hosts.

In malaria systems, manipulation is coordinated with the development of the parasite to maximize transmission. Mosquitoes carrying the transmissible sporozoite stage are more motivated to bite the vertebrate host than mosquitoes carrying the non-transmissible oocyst stage [19,20]. Similarly, mosquitoes are more attracted to vertebrate hosts carrying the transmissible gametocyte stage than to hosts carrying the non-transmissible asexual stage [21,24]. In contrast to malaria parasites, *Borrelia* spirochetes do not go through a sequence of developmental stages that differ in transmissibility. We therefore do not expect that the age of the *Borrelia* infection inside the nymph would influence the manipulation.

To test whether tick-borne pathogens can manipulate tick host choice behaviour requires a good understanding of this tick phenotype. The host choice behaviour of *I. ricinus* ticks has not received a lot of study. Immature *I. ricinus* ticks use different hosts across Europe: rodents and birds in Switzerland [27,65], birds but rarely rodents in the British Isles [66-68], and lizards in southern Europe and North Africa [69,70]. Recent genetic studies on *I. ricinus* suggest that this tick species might have differentiated into races that have a preference for certain host species [71]. To date, no study has demonstrated whether European populations of *I. ricinus* have evolved preferences for locally available hosts. The host choice behaviour of other *Ixodes* ticks has received more attention [8,72,73]. Basic knowledge of tick host choice behaviour is critical for studying whether tick-borne pathogens can manipulate this phenotype.

## Conclusion

In summary, our study found no evidence that infection with *Borrelia* pathogens influenced the attraction of *I. ricinus* nymphs to rodent odours under laboratory conditions. *Borrelia* pathogens may influence other aspects of tick host choice behaviour such as the probability of rejecting a host following attachment. Host choice is a matter of life and death for *Borrelia burgdorferi* s. l. and this pathogen would clearly benefit by manipulating the tick to reject incompetent vertebrate hosts. Future studies of whether tick-borne pathogens can manipulate tick host choice behaviour will improve our understanding of the ecology of ticks and tick-borne diseases.

## Additional file

Additional file 1: Table S1. Results of the tick questing behaviour trials for trial types A, B, and C. For each trial we show the total number of ticks at the start of the trial (n.start), the number of ticks that remained in the system at the end of the trial (n.total), the number of ticks that remained in the system and that climbed a questing perch (n.active), the number of active ticks that chose the scented questing perch (n.choice), the proportion of active ticks that chose the scented questing perch (p.choice), the exact binomial probability that random chance produced the observed number of ticks on the scented questing perch (p.value), and whether this probability was < 0.05 or not (signif).

## References

[1] Gern L, Humair P (2002). Ecology of Borrelia burgdorferi sensu lato in Europe. Lyme Borreliosis Biology, Epidemiology and Control. CABI Int.

[2] Hoogstraal H, Aeschlimann A (1982). Tick-host specificity. Bulletin de la société entomologique suisse.

[3] Keirans JE, Hutcheson H, Durden LA, Klompen J (1996). *Ixodes* (Ixodes) *scapularis* (Acari: Ixodidae): redescription of all active stages, distribution, hosts, geographical variation, and medical and veterinary importance. J Med Entomol.

[4] McCoy KD, Léger E, Dietrich M (2013). Host specialization in ticks and transmission of tick-borne diseases: a review. Front Cell Infect Microbiol.

[5] Ostfeld RS, Keesing F (2000). Biodiversity series: the function of biodiversity in the ecology of vector-borne zoonotic diseases. Can J Zool.

[6] LoGiudice K, Ostfeld RS, Schmidt KA, Keesing F (2003). The ecology of infectious disease: effects of host diversity and community composition on Lyme disease risk. Proc Natl Acad Sci.

[7] Casher L, Lane R, Barrett R, Eisen L (2002). Relative importance of lizards and mammals as hosts for ixodid ticks in northern California. Exp Appl Acarol.

[8] Slowik TJ, Lane RS (2009). Feeding preferences of the immature stages of three western North American ixodid ticks (Acari) for avian, reptilian, or rodent hosts. J Med Entomol.

[9] Salkeld DJ, Lane RS (2010). Community ecology and disease risk: lizards, squirrels, and the Lyme disease spirochete in California, USA. Ecology.

[10] Lane RS, Quistad G (1998). Borreliacidal factor in the blood of the western fence lizard (Sceloporus occidentalis). J Parasitol.

[11] Lefèvre T, Koella JC, Renaud F, Hurd H, Biron DG, Thomas F (2006). New prospects for research on manipulation of insect vectors by pathogens. PLoS Pathog.

[12] Lefèvre T, Thomas F (2008). Behind the scene, something else is pulling the strings: emphasizing parasitic manipulation in vector-borne diseases. Infect Genet Evol.

[13] Hurd H (2003). Manipulation of medically important insect vectors by their parasites. Annu Rev Entomol.

[14] Moore J (1993). Parasites and the behavior of biting flies. J Parasitol.

[15] Beach R, Kiilu G, Leeuwenburg J (1985). Modification of sand fly biting behavior by *Leishmania* leads to increased parasite transmission. Am J Trop Med Hyg.

[16] Wekesa JW, Copeland RS, Mwangi RW (1992). Effect of *Plasmodium falciparum* on blood feeding behavior of naturally infected *Anopheles* mosquitoes in western Kenya. Am J Trop Med Hyg.

[17] Rossignol P, Ribeiro J, Spielman A (1984). Increased intradermal probing time in sporozoite-infected mosquitoes. Am J Trop Med Hyg.

[18] Koella JC, SÖrensen FL, Anderson R (1998). The malaria parasite, *Plasmodium falciparum*, increases the frequency of multiple feeding of its mosquito vector, *Anopheles gambiae*. Proc Roy Soc Lond B Biol Sci.

[19] Anderson RA, Koellaf J, Hurd H (1999). The effect of *Plasmodium yoeliinigeriensis* infection on the feeding persistence of *Anopheles stephensi* Liston throughout the sporogonic cycle. Proc Roy Soc Lond B Biol Sci.

[20] Koella JC, Rieu L, Paul RE (2002). Stage-specific manipulation of a mosquito’s host-seeking behavior by the malaria parasite *Plasmodium gallinaceum*. Behav Ecol.

[21] Lacroix R, Mukabana WR, Gouagna LC, Koella JC (2005). Malaria infection increases attractiveness of humans to mosquitoes. PLoS Biol.

[22] O'Shea B, Rebollar-Tellez E, Ward R, Hamilton J, El Naiem D, Polwart A (2002). Enhanced sandfly attraction to *Leishmania*-infected hosts. Trans R Soc Trop Med Hyg.

[23] Cornet S, Nicot A, Rivero A, Gandon S (2013). Malaria infection increases bird attractiveness to uninfected mosquitoes. Ecol Lett.

[24] De Moraes CM, Stanczyk NM, Betz HS, Pulido H, Sim DG, Read AF, Mescher MC (2014). Malaria-induced changes in host odors enhance mosquito attraction. Proc Natl Acad Sci.

[25] Meiners T, Werkhausen A, Nierhaus L, Dautel H (2011). Infection of ticks with Borrelia afzelii cuts of olfactory orientation towards certain host kairomones. XI International Jena Symposium on Tick-Borne Diseases.

[26] Vollandt D, Ruzek D, Dautel H, Meiners T, Niedrig M (2011). Infection with tick-borne encephalitis virus changes responses of Ixodes ricinus nymphs and adults to mammal odours. XI International Jena Symposium on Tick-Borne Diseases.

[27] Piesman J, Gern L (2004). Lyme borreliosis in Europe and North America. Parasitology.

[28] Hanincová K, Schäfer S, Etti S, Sewell H-S, Taragelova V, Ziak D, Labuda M, Kurtenbach K (2003). Association of *Borrelia afzelii* with rodents in Europe. Parasitology.

[29] Kurtenbach K, Peacey M, Rijpkema SG, Hoodless AN, Nuttall PA, Randolph SE (1998). Differential transmission of the genospecies of *Borrelia burgdorferi* sensu lato by game birds and small rodents in England. Appl Environ Microbiol.

[30] Kurtenbach K, Sewell H-S, Ogden NH, Randolph SE, Nuttall PA (1998). Serum complement sensitivity as a key factor in Lyme disease ecology. Infect Immun.

[31] Kurtenbach K, De Michelis S, Etti S, Schäfer SM, Sewell H-S, Brade V, Kraiczy P (2002). Host association of *Borrelia burgdorferi* sensu lato–the key role of host complement. Trends Microbiol.

[32] Huegli D, Hu C, Humair P-F, Wilske B, Gern L (2002). *Apodemus* species mice are reservoir hosts of *Borrelia garinii* OspA serotype 4 in Switzerland. J Clin Microbiol.

[33] Humair P-F, Peter O, Wallich R, Gern L (1995). Strain variation of Lyme disease spirochetes isolated from *Ixodes ricinus* ticks and rodents collected in two endemic areas in Switzerland. J Med Entomol.

[34] Humair P-F, Gern L (1998). Relationship between *Borrelia burgdorferi* sensu lato species, red squirrels (*Sciurus vulgaris*) and *Ixodes ricinus* in enzootic areas in Switzerland. Acta Trop.

[35] Hanincová K, Taragelová V, Koci J, Schäfer SM, Hails R, Ullmann AJ, Piesman J, Labuda M, Kurtenbach K (2003). Association of *Borrelia garinii* and *B. valaisiana* with songbirds in Slovakia. Appl Environ Microbiol.

[36] Heylen D, Matthysen E, Fonville M, Sprong H (2014). Songbirds as general transmitters but selective amplifiers of *Borrelia burgdorferi* sensu lato genotypes in *Ixodes rinicus* ticks. Environ Microbiol.

[37] Humair P-F, Postic D, Wallich R, Gern L (1998). An avian reservoir (*Turdus merula*) of the Lyme borreliosis spirochetes. Zentralblatt für Bakteriologie.

[38] Kurtenbach K, Schäfer SM, Sewell H-S, Peacey M, Hoodless A, Nuttall PA, Randolph SE (2002). Differential survival of Lyme borreliosis spirochetes in ticks that feed on birds. Infect Immun.

[39] Lommano E, Dvořák C, Vallotton L, Jenni L, Gern L (2014). Tick-borne pathogens in ticks collected from breeding and migratory birds in Switzerland. Ticks and Tick-Borne Diseases.

[40] Norte A, Ramos J, Gern L, Núncio M, Lopes de Carvalho I (2013). Birds as reservoirs for *Borrelia burgdorferi* s.l. in Western Europe: circulation of *B. turdi* and other genospecies in bird–tick cycles in Portugal. Environ Microbiol.

[41] Norte AC, Lopes de Carvalho I, Núncio MS, Ramos JA, Gern L (2013). Blackbirds *Turdus merula* as competent reservoirs for *Borrelia turdi* and *Borrelia valaisiana* in Portugal: evidence from a xenodiagnostic experiment. Environ Microbiol Rep.

[42] Taragel'ová V, Koči J, Hanincová K, Kurtenbach K, Derdáková M, Ogden NH, Literák I, Kocianová E, Labuda M (2008). Blackbirds and song thrushes constitute a key reservoir of *Borrelia garinii*, the causative agent of borreliosis in Central Europe. Appl Environ Microbiol.

[43] Crooks E, Randolph SE (2006). Walking by *Ixodes ricinus* ticks: intrinsic and extrinsic factors determine the attraction of moisture or host odour. J Exp Biol.

[44] QIAGEN. Purification of total DNA from ticks using the DNeasy® Blood & Tissue Kit for detection of *Borrelia* DNA (DY16 Jun-08). In*.* QIAGEN Supplementary Protocol; 2008.

[45] Schwaiger M, Peter O, Cassinotti P (2001). Routine diagnosis of *Borrelia burgdorferi* (sensu lato) infections using a real‐time PCR assay. Clin Microbiol Infect.

[46] Wallich R, Moter S, Simon M, Ebnet K, Heiberger A, Kramer M (1990). The *Borrelia burgdorferi* flagellum-associated 41-kilodalton antigen (flagellin): molecular cloning, expression, and amplification of the gene. Infect Immun.

[47] Alekseev AN, Dubinina HV, Van De Pol I, Schouls LM (2001). Identification of *Ehrlichia* spp. and *Borrelia burgdorferi* in *Ixodes* ticks in the Baltic regions of Russia. J Clin Microbiol.

[48] Herrmann C, Gern L (2010). Survival of *Ixodes ricinus* (Acari: Ixodidae) under challenging conditions of temperature and humidity is influenced by *Borrelia burgdorferi* sensu lato infection. J Med Entomol.

[49] Richter D, Postic D, Sertour N, Livey I, Matuschka F-R, Baranton G (2006). Delineation of *Borrelia burgdorferi* sensu lato species by multilocus sequence analysis and confirmation of the delineation of *Borrelia spielmanii* sp. nov. Int J Syst Evol Microbiol.

[50] Madden T. The BLAST Sequence Analysis Tool. *The NCBI Handbook [Internet]* 2002, Chapter 16(Bethesda (MD): National Center for Biotechnology Information (US)).

[51] RStudio. RStudio: Integrated development environment for R (Version 0.98.1102) [Computer software]. 2014, http://www.rstudio.org/.

[52] Cadenas FM, Rais O, Humair P-F, Douet V, Moret J, Gern L (2007). Identification of host bloodmeal source and *Borrelia burgdorferi* sensu lato in field-collected *Ixodes ricinus* ticks in Chaumont (Switzerland). J Med Entomol.

[53] Herrmann C, Gern L (2013). Survival of *Ixodes ricinus* (Acari: *Ixodidae*) nymphs under cold conditions is negatively influenced by frequent temperature variations. Ticks and Tick-Borne Diseases.

[54] Herrmann C, Gern L (2012). Do the level of energy reserves, hydration status and *Borrelia* infection influence walking by *Ixodes ricinus* (Acari: Ixodidae) ticks?. Parasitology.

[55] Herrmann C, Voordouw M, Gern L (2013). *Ixodes ricinus* ticks infected with the causative agent of Lyme disease, *Borrelia burgdorferi* sensu lato, have higher energy reserves. Int J Parasitol.

[56] Lefcort H, Durden L (1996). The effect of infection with Lyme disease spirochetes (*Borrelia burgdorferi*) on the phototaxis, activity, and questing height of the tick vector *Ixodes scapularis*. Parasitology.

[57] Romashchenko AV, Ratushnyak AS, Zapara TA, Tkachev SE, Moshkin MP (2012). The correlation between tick (*Ixodes persulcatus* Sch.) questing behaviour and synganglion neuronal responses to odours. J Insect Physiol.

[58] Herrmann C, Gern L (2015). Search for blood or water is influenced by *Borrelia burgdorferi* in *Ixodes ricinus*. Parasites & Vectors.

[59] Waladde S, Rice M, Obenchain FD, Galun R (1982). The sensory basis of tick feeding behaviour. The Physiology of Ticks.

[60] Carroll J, Mills G, Schmidtmann E (1996). Field and laboratory responses of adult *Ixodes scapularis* (Acari: *Ixodidae*) to kairomones produced by white-tailed deer. J Med Entomol.

[61] Carroll J, Klun J, Schmidtmann E (1995). Evidence for kairomonal influence on selection of host-ambushing sites by adult *Ixodes scapularis* (Acari: *Ixodidae*). J Med Entomol.

[62] Carroll J (1999). Notes on responses of blacklegged ticks (Acari: *Ixodidae*) to host urine. J Med Entomol.

[63] Carroll J (2000). Responses of adult *Ixodes scapularis* (Acari: *Ixodidae*) to urine produced by white-tailed deer of various reproductive conditions. J Med Entomol.

[64] Lees A (1948). The sensory physiology of the sheep tick, *Ixodes ricinus* L. J Exp Biol.

[65] Gern L (2004). Tiques et borréliose de Lyme en Suisse occidentale. Bull Soc Neuchateloise Scie Nat.

[66] Ogden N, Nuttall P, Randolph S (1997). Natural Lyme disease cycles maintained via sheep by co-feeding ticks. Parasitology.

[67] Pichon B, Rogers M, Egan D, Gray J (2005). Blood-meal analysis for the identification of reservoir hosts of tick-borne pathogens in Ireland. Vector Borne Zoonotic Dis.

[68] Harrison A, Montgomery W, Bown K (2011). Investigating the persistence of tick-borne pathogens via the R0 model. Parasitology.

[69] Ekner A, Dudek K, Sajkowska Z, Majláthová V, Majláth I, Tryjanowski P (2011). *Anaplasmataceae* and *Borrelia burgdorferi* sensu lato in the sand lizard *Lacerta agilis* and co-infection of these bacteria in hosted *Ixodes ricinus* ticks. Parasites & Vectors.

[70] De Sousa R, de Carvalho IL, Santos A, Bernardes C, Milhano N, Jesus J, Menezes D, Núncio M (2012). Role of the lizard *Teira dugesii* as a potential host for *Ixodes ricinus* tick-borne pathogens. Appl Environ Microbiol.

[71] Kempf F, De Meeûs T, Vaumourin E, Noel V, Taragel’ová V, Plantard O, Heylen DJ, Eraud C, Chevillon C, McCoy KD (2011). Host races in *Ixodes ricinus*, the European vector of Lyme borreliosis. Infect Genet Evol.

[72] Shaw MT, Keesing F, McGrail R, Ostfeld RS (2003). Factors influencing the distribution of larval blacklegged ticks on rodent hosts. Am J Trop Med Hyg.

[73] Swei A, Ostfeld RS, Lane RS, Briggs CJ (2011). Impact of the experimental removal of lizards on Lyme disease risk. Proc Biol Sci.

