# Supplementary material for: Lyme disease bacterium does not affect attraction to rodent odour in the tick vector
Source: Parasit Vectors. 2015 Apr 28;8:249. doi: 10.1186/s13071-015-0856-8 (PMC4417542; doi:10.1186/s13071-015-0856-8)
Supplement: Additional file 1: Table S1. — Results of the tick questing behaviour trials for trial types A, B, and C. For each trial we show the total number of ticks at the start of the trial (n.start), the number of ticks that remained in the system at the end of the trial (n.total), the number of ticks that remained in the system and that climbed a questing perch (n.active), the number of active ticks that chose the scented questing perch (n.choice), the proportion of active ticks that chose the scented questing perch (p.choice), the exact binomial probability that random chance produced the observed number of ticks on the scented questing perch (p.value), and whether this probability was < 0.05 or not (signif). [file 13071_2015_856_MOESM1_ESM.docx]

**Additional file 1**

**Table S1. Results of the tick questing behaviour trials for trial types A, B, and C.**

For each trial we show the total number of ticks at the start of the trial (n.start), the number of ticks that remained in the system at the end of the trial (n.total), the number of ticks that remained in the system and that climbed a questing perch (n.active), the number of active ticks that chose the scented questing perch (n.choice), the proportion of active ticks that chose the scented questing perch (p.choice), the exact binomial probability that random chance produced the observed number of ticks on the scented questing perch (p.value), and whether this probability was < 0.05 or not (signif).

| Trial | Type | n.start | n.total | n.active | n.choice | p.choice | p.value | signif |
| --- | --- | --- | --- | --- | --- | --- | --- | --- |
| 9 | A | 20 | 16 | 16 | 1 | 0.063 | 0.612 | 0 |
| 1 | A | 20 | 19 | 12 | 1 | 0.083 | 0.453 | 0 |
| 7 | A | 20 | 20 | 18 | 2 | 0.111 | 0.395 | 0 |
| 5 | A | 20 | 14 | 8 | 1 | 0.125 | 0.264 | 0 |
| 2 | A | 20 | 15 | 7 | 1 | 0.143 | 0.215 | 0 |
| 6 | A | 20 | 16 | 10 | 2 | 0.200 | 0.361 | 0 |
| 10 | A | 20 | 17 | 14 | 3 | 0.214 | 0.087 | 0 |
| 8 | A | 20 | 15 | 13 | 4 | 0.308 | 0.016 | 1 |
| 4 | A | 20 | 14 | 11 | 4 | 0.364 | 0.007 | 1 |
| 3 | A | 20 | 13 | 8 | 5 | 0.625 | 0.0001 | 1 |
| 18 | B | 20 | 14 | 1 | 0 | 0.000 | 0.125 | 0 |
| 22 | B | 20 | 15 | 8 | 0 | 0.000 | 0.656 | 0 |
| 25 | B | 20 | 14 | 9 | 0 | 0.000 | 0.699 | 0 |
| 26 | B | 20 | 17 | 10 | 0 | 0.000 | 0.737 | 0 |
| 11 | B | 20 | 13 | 10 | 1 | 0.100 | 0.361 | 0 |
| 16 | B | 20 | 10 | 8 | 1 | 0.125 | 0.264 | 0 |
| 15 | B | 20 | 16 | 7 | 2 | 0.286 | 0.046 | 1 |
| 12 | B | 20 | 15 | 11 | 4 | 0.364 | 0.007 | 1 |
| 27 | B | 20 | 16 | 9 | 4 | 0.444 | 0.002 | 1 |
| 30 | B | 20 | 11 | 9 | 5 | 0.556 | 0.0002 | 1 |
| 17 | C | 20 | 12 | 5 | 0 | 0.000 | 0.487 | 0 |
| 20 | C | 20 | 12 | 8 | 0 | 0.000 | 0.656 | 0 |
| 21 | C | 17 | 12 | 6 | 0 | 0.000 | 0.551 | 0 |
| 13 | C | 20 | 10 | 8 | 1 | 0.125 | 0.264 | 0 |
| 29 | C | 20 | 14 | 10 | 2 | 0.200 | 0.119 | 0 |
| 14 | C | 20 | 14 | 8 | 2 | 0.250 | 0.067 | 0 |
| 19 | C | 20 | 18 | 11 | 3 | 0.273 | 0.039 | 1 |
| 24 | C | 20 | 12 | 6 | 2 | 0.333 | 0.029 | 1 |
| 28 | C | 20 | 12 | 9 | 3 | 0.333 | 0.018 | 1 |
| 23 | C | 20 | 12 | 8 | 3 | 0.375 | 0.011 | 1 |
